# Novel cholesterol lowering drugs: Can phase 2/3 clinical trial safety assessments predict cardiovascular event outcome trial efficacy?

**DOI:** 10.1016/j.ahjo.2026.100728

**Published:** 2026-01-28

**Authors:** Charles Shear, Michael H. Davidson, Marc Ditmarsch, John J.P. Kastelein, Michael Szarek

**Affiliations:** a no current affiliation-biopharmaceutical consultant, Collegeville, United States of America; b NewAmsterdam Pharma, Amsterdam, Netherlands; c Department of Vascular Medicine, University of Amsterdam, Amsterdam, Netherlands; d Mount Sinai Fuster Heart Hospital, Icahn School of Medicine, Mount Sinai, New York, United States of America; e University of Colorado Anschutz Medical Campus, Aurora, United States of America; f State University of New York Downstate School of Public Health, New York, United States of America

## Abstract

**Setting:**

Prior to a cardiovascular outcomes trial (CVOT), novel cholesterol-lowering therapies undergo phase 2/3 studies for their lipid and atherosclerotic effects and safety (non-CVOTs). Since the occurrence of major adverse cardiovascular events (MACE) is part of the safety assessment, nominal reductions or increases may be observed prior to definitive testing of the effect in a CVOT.

**Study objective:**

To investigate if the observed MACE treatment effect in non-CVOT lipid-lowering registration studies holds value in predicting the outcome in a CVOT trial, typically reported later than the initial lipid-lowering studies.

**Design/participants/interventions:**

We reviewed recent development programs for cholesterol-lowering drugs that had completed non-CVOT and CVOT studies. MACE data were compared for phase 2/3 non-CVOT versus pivotal CVOT results.

**Main outcome measures:**

Our primary outcome was a qualitative comparison for directionally concordant consistency in MACE risk ratio treatment effects (harm, neutrality, or benefit). Correlation analysis was also performed.

**Results:**

Seven drugs were reviewed in 3 cholesterol-lowering classes: CETP inhibitors, bempedoic acid, and PCSK9 inhibitors. Concordance in non-CVOT vs CVOT results was seen in 6 of 7 drugs. One drug (dalcetrapib) had a trend for benefit observed, albeit with very small numbers, in early development, but showed a neutral CVOT. There was a moderate correlation between the risk reductions or increases from the non-CVOTs and CVOTs: *r* = 0.69, *p* = 0.0893.

**Conclusion:**

Within the limitations of the drugs studied and the variability in MACE definitions, there is value in the results of non-CVOTs to predict the CVOT outcome.

## Graphical abstract

**Figure:**
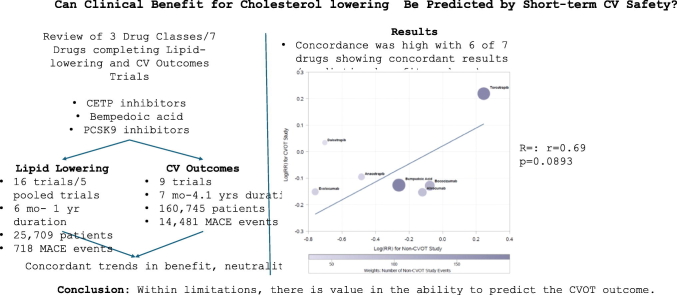

## Abbreviations

**Table:** 

ApoC3	apolipoprotein C3
CETP	cholesterol ester transfer protein
CV	cardiovascular
CVOT	cardiovascular outcomes trial
HR	hazard ratio
Lp(a)	lipoprotein (a) inhibitor
MACE	major adverse cardiovascular events
PCSK9	proprotein convertase subtilisin/kexin type 9
RR	risk ratio

## Keywords

Cholesterol-lowering drugs

Cardiovascular risk

CETP inhibitors

PCSK9

Bempedoic acid

Lp (a) inhibitors

## 1 Introduction

Since the 1950s landmark advancements in the treatment of hypercholesterolemia and prevention of atherosclerotic cardiovascular (CV) disease have occurred. Medical therapeutics have gone from the multigram dosing of bile acid sequestrants and its landmark LRC CPPT trial [1] to the advent of small molecule statin drugs, ezetimibe, bempedoic acid, and inhibitors of PCSK9, CETP, Lp(a) and ApoC3 [2]. The development pathway for novel hypolipidemic drugs has become well established and typically includes phase 1/2 pharmacokinetic/pharmacodynamic and proof-of-concept studies followed by dose-ranging lipid and atherosclerotic imaging trials (referred to hereafter as non-CV outcomes trials (CVOTs)). Drugs can then either progress to confirmatory non-CVOTs in phase 3 and its corresponding safety database or, alternatively for novel mechanisms, a CVOT can be initiated and replace or supplement non-CVOTs when needed for regulatory approval as seen with novel Lp(a) drugs [3]. Safety database sizes and duration of exposure are typically driven by regulatory considerations and the particular characteristics of the drug in development. As adopted by most regulatory agencies, The International Conference on Harmonization E1 guidance states that for long-term treatment, a drugs' safety database should have at least 1500 patients exposed for any length of time, 300–600 patients for 6 months, and at least 100 patients for 12 months to properly evaluate safety [4].

As the non-CVOTs involve markedly smaller sample sizes and shorter durations than CVOTs, those results are typically known years ahead of the completion of a CVOT. Recently, a pooled analysis of phase 3 lipid trials showed a significant reduction in major adverse cardiovascular events (MACE) by obicetrapib after 6 months of treatment [5]. In accompanying editorials it was noted that this was a promising signal needing confirmation [6] as the studies were not designed to assess cardiovascular outcomes [7]. We wondered if the obicetrapib experience was just a fortunate ‘luck-of-the-draw’ or if other contemporary cholesterol-lowering drugs experienced a trend in benefit or harm in MACE when comparing the phase 2/3 lipid trial safety data to the later reported CVOT.

We therefore asked the following study question: Do CV safety data from non-CVOTs, which are typically underpowered for an evaluation of CV event efficacy, help predict the efficacy outcome of the CVOT? If so, the results could help stakeholders in future development program decisions about progression to executing a CVOT, or about design features of a CVOT that is underway. We believe this is the first systematic evaluation of this study question.

## 2 Materials and methods

### 2.1 Survey of the literature for phase 2/3 trials for contemporary lipid lowering drug programs

We used Pubmed, Embase, MEDLINE, ClinicalTrials.gov, and the Cochrane Library, covering the last 20-year timeframe period to identify cholesterol-lowering eligible drug programs and their clinical trials. The resulting classes of drugs that had reached advanced stages of clinical development independent of their registration (NDA/BLA) approval status included: CETP inhibitors, bempedoic acid, and PCSK9 inhibitors. Excluded from the review were fibrates as they are not typically used for first line treatment of hypercholesterolemia. Trial eligibility criteria were as follows: data were analyzed from randomized clinical trials that compared an active drug or a drug combination with a comparator (placebo or the combination add-on). In addition, participants were 18 years and older; the trial enrolled at least 100 participants; the active treatment period was at least 24 weeks; and the study reported at least one measure of MACE outcome. Based on this, ezetimibe was also excluded as the only non-CVOT data with more than 12 weeks of treatment was uncontrolled and open label in design [8].

We compared the overall non-CVOT MACE phase 2/3 safety data for a drug and compared it to the result of the primary MACE outcome in its CVOT. Our primary outcome was a qualitative one to see if there was concordance in the results: MACE benefit, MACE neutrality, or MACE harm.

When multiple sources of data for a program/trial existed, we used the most comprehensive data set, typically the FDA Medical Review of the corresponding drug's pooled trail sets as the sole source of non-CVOT data. The primary publication of the CVOT was used as its source of data.

### 2.2 Analysis

We compared the published study level CVOT primary outcome risk reduction estimate from the literature and compared it to the study level non-CVOT MACE safety data incidence of MACE for the drug under review and its comparator. For the latter, we used as a summary measure either pooled analyses from the corresponding FDA Medical review (if available) or in its absence, by combining the trials deemed eligible for a drug to provide a measure of its MACE safety performance. Although there was general consistency in the included MACE components, there was no attempt to standardize the MACE composite definition used across studies.

We then provide a global measure of concordance of CVOT vs non-CVOT MACE performance by categorizing the result as either: benefit, neutral or harm. This was determined as follows: 1) for CVOTs, the statistical significance of the primary MACE treatment effect was used to define benefit, neutrality or harm and, 2) for non-CVOTs, the directional imbalance of the numeric MACE treatment effect indicating either potential harm, neutrality, or potential benefit. Statistical significance was not used in this non-CVOT determination.

Treatment effects on MACE in non-CVOTs were estimated by risk ratios (RRs) and corresponding 95% confidence intervals (CIs) based on the observed proportions in each treatment group who experienced an event. Treatment effects on MACE in CVOTs were the published hazard ratios (HRs) and corresponding 95% CIs.

The relationships between CVOT and non-CVOT treatment effects were also analyzed by weighted correlation and linear regression analyses of the log(*RR*)s vs. log(HRs). In the regression analysis, the non-CVOT log(RR) was the predictor and the CVOT log(HR) was the outcome. Weights were determined by the number of MACE events in the non-CVOTs. The slope of the regression line and its corresponding 95% CI was also estimated.

## 3 Results

### 3.1 Trials identified/used in analysis

In all, 9 CVOT trials were identified for potential use in the analysis. These trials included 160,745 patients and 14,481 MACE events with a mean or median length of follow-up for their primary results ranging from 7 months (bococizumab, terminated early) to 4.1 years (anacetrapib, HPS3/TIMI 55). For non-CVOT trials, 16 individual studies and 5 trial pools were identified. This included 25,709 patients and 718 MACE events with a length of follow-up less than 1 year. Table 1 displays a summary of the trials used in the analysis. Supplemental Table 1 provides a more detailed listing of each trial considered and their disposition in the analysis.

Given the paucity of evacetrapib non-CVOT data, we enhanced the scope of eligible studies with the short-term phase 3 Japanese population double-blind data (3 months) as well as the ACCENTUATE open label extension. Still, the data were insufficient (1 event in each of the double-blind and OLE studies) to evaluate further and were not used. For bempedoic acid, the No/low statin Pool (trials 1002–046 and 1002–048) as defined by FDA were excluded as the MACE data have not been reported for that group. Also excluded from analysis was the secondary 6.4-year follow-up of the HPS3/TIMI 55 trial as it was a safety assessment associated with the long half-life of anacetrapib in adipose tissue [9].

**Table 1:** Clinical trials summary used in the analysis.

Drug/ Reference*	Study Type		Sample Size and f/u			MACE INCLUSIONS						
	CVOT	Non-CVOT	N- active	N- comp.	Follow-up Length	CHD death	CVD death	NF-MI	NF-stroke	Revasc	Hosp U/A/ ACS	other
***CETP Inhibitor: Evacetrapib***												
CVOT: Lincoff et al. 2017 [18]	x		6038	6054	2.3 yrs		x	x	x	x(any)	x	
Non-CVOT Summary [19], [20], [21], [22]		x	450	366	3–12 mo	No standardized definition across studies						
***CETP Inhibitor: Anacetrapib***												
CVOT: HPS3/TIMI55–REVEAL Collab Grp 2017 [23]	x		15,225	15,224	4.1 yrs	x		x		x(any)		
Non-CVOT Summary [24], [25], [26], [27], [28]		x	1810	1455	3–18 mo		x	x	x		x	
***CETP Inhibitor: Dalcetrapib***												
CVOT: Schwartz et al. 2012 [29]	x		7938	7933	2.6 yrs	x		x	x(isch)		x	c. arrest
Non CVOT Summary [30], [31], [32]		x	517	375	3–24 mo	x		x	x(isch)	x(any)	x	
***CETP Inhibitor: Torcetrapib + atorvastatin***												
CVOT: Barter et al. 2007 [33]	x		7533	7534	1.5 yrs	x		x	x		x	
Non-CVOT Summary [34], [35], [36]		x	1418	1426	22–24 mo	No standardized definition across studies						
***Bempedoic Acid***												
CVOT: Nissen et al. 2023 [37]	x		6992	6978	3.4 yrs		x	x	x	x(any)		
Non-CVOT Summary [38]		x	2424	1197	12 mo		x	x	x	x	x	
***PCSK9 Inhibitor: Evolocumab***												
CVOT: Sabatine et al., 2017 [39]	x		13,784	13,780	2.2 yrs		x	x	x	x(any)	x	
Non-CVOT Summary [40]		x	2976	1489	11.1 mo	x		x	x	x(any)	x	
***PCSK9 Inhibitor: Alirocumab***												
CVOT: Schwartz et al. 2018 [41]	x		9462	9462	2.8 yrs	x	x(isch)	x	x(isch)		x	
Non-CVOT Summary [42]		x	3182	1792	6–15.1 mo	x	x(isch)	x	x(isch)		x	
***PCSK9 Inhibitor: Bococizumab***												
CVOT SPIRE-1 and SPIRE-2: Ridker et al. 2017a [43]	x		13,720	13,718	0.8 yrs		x	x	x	x		
Non-CVOT Summary [44]		x	2377	2058	3–12 mo		x	x	x	x		

*Complete reference information for studies used and not used in CVOT and NON-CVOT Summary can be found in supplementary Table 1.

*Complete reference information for studies used and not used in CVOT and NON-CVOT Summary can be found in supplementary Table 1.

CHD = coronary heart disease death; CVD = cardiovascular disease death; comp. = comparator; NF-MI = non-fatal myocardial infarction; NF-stroke = non-fatal stroke; revasc = urgent coronary revascularization unless specified as any; Hosp U/A/ ACS = hospitalization for unstable angina or acute coronary syndrome; c. arrest = cardiac arrest.

In all, 7 drugs in development were reviewed in 3 different mechanism-of action classes. Concordance was high with 6 of 7 drugs showing concordant results (predicting benefit or harm; see Table 2).

**Table 2:** Concordance results for lipid lowering drugs evaluated.

Drug	Sample Size		MACE EVENTS (1st occurrence)				CVOT 1^0^ MACE HR (95% CI) Non-CVOT MACE RR (95% CI)	Concordance
	N-active drug	N-comparator	Events-Drug	Events-Comparator	% Event Rate-Drug	% Event Rate-Comparator		
**Evacetrapib-CVOT**	6038	6054	779	776	12.90	12.82	**1.01 (0.91–1.11)**	not evaluated*
**Non-CVOT**	450	366	1	1	0.22	0.27	**0.81 (0.05–12.96)**	
**Anacetrapib-CVOT**	15,225	15,224	1640	1803	10.77	11.84	**0.91 (0.85–0.97)**	**Concordant**
Non-CVOT	1810	1455	23	30	1.27	2.06	**0.61 (0.36–1.06)**	
**Dalcetrapib-CVOT**	7938	7933	656	633	8.26	7.98	**1.04 (0.93–1.16)**	**Not concordant**
Non CVOT	517	375	13	19	2.51	5.07	**0.50 (0.25–0.99)**	
**Torcetrapib-CVOT**	7533	7534	464	373	6.16	4.95	**1.25 (1.09–1.44)**	**Concordant**
Non-CVOT	1418	1426	103	81	7.26	5.68	**1.28 (0.96–1.70)**	
**Bempedoic Acid-CVOT**	6992	6978	819	927	11.71	13.28	**0.87 (0.79–0.96)**	**Concordant**
Non-CVOT	2424	1197	117	75	4.83	6.27	**0.77 (0.58–1.02)**	
**Evolocumab-CVOT**	13,784	13,780	1344	1563	9.75	11.34	**0.85 (0.79–0.92)**	**Concordant**
Non-CVOT	2976	1489	28	30	0.94	2.01	**0.47 (0.28–0.78)**	
**Alirocumab-CVOT**	9462	9462	903	1052	9.54	11.12	**0.85 (0.78–0.93)**	**Concordant**
Non-CVOT	3182	1792	52	33	1.63	1.84	**0.89 (0.57–1.37)**	
**Bococizumab-CVOT**	13,720	13,718	352	397	3.16	3.59	**0.88 (0.76–1.02)**	**Concordant**
Non-CVOT	2377	2058	57	55	2.5	2.7	**0.90 (0.62–1.29)**	

*see text for explanation; CVOT = cardiovascular outcomes trial; MACE = major cardiovascular event; HR = hazard ratio; RR = risk ratio; atorva = atorvastatin.

Complete reference information for studies used and not used in CVOT and Non-CVOT summary can be found in supplementary Table 1.

Complete reference information for studies used and not used in CVOT and Non-CVOT summary can be found in supplementary Table 1.

Potential harm as indicated by an imbalance in non-CVOT trial events (RR (95% CI) = 1.28 (0.96, 1.70)) was only observed with torcetrapib. This was predictive of the CVOT outcome which was terminated early for increased MACE of a similar magnitude (HR (95% CI) = 1.25 (1.09, 1.44)).

Potential benefit as indicated by a clear imbalance (RR ≤ 0.90) in favor of the drug for non-CVOT trials was evident for anacetrapib, bempedoic acid, evolocumab, alirocumab, and bococizumab. These results were also predictive of the CVOT beneficial outcome. In contrast, the dalcetrapib program non-CVOTs predicted a positive outcome (RR (95% CI) = 0.50 (0.25, 0.99)) for its CVOT trial but the later produced a neutral result (HR (95% CI) = 1.04 (0.93, 1.16). Of note, the signal for benefit in the non-CVOTs was based on only 32 events, which was the smallest number of any of the non-CVOTs that were considered, and the resulting wide confidence interval included the possibility of a minimal treatment benefit.

**Fig. 1:**
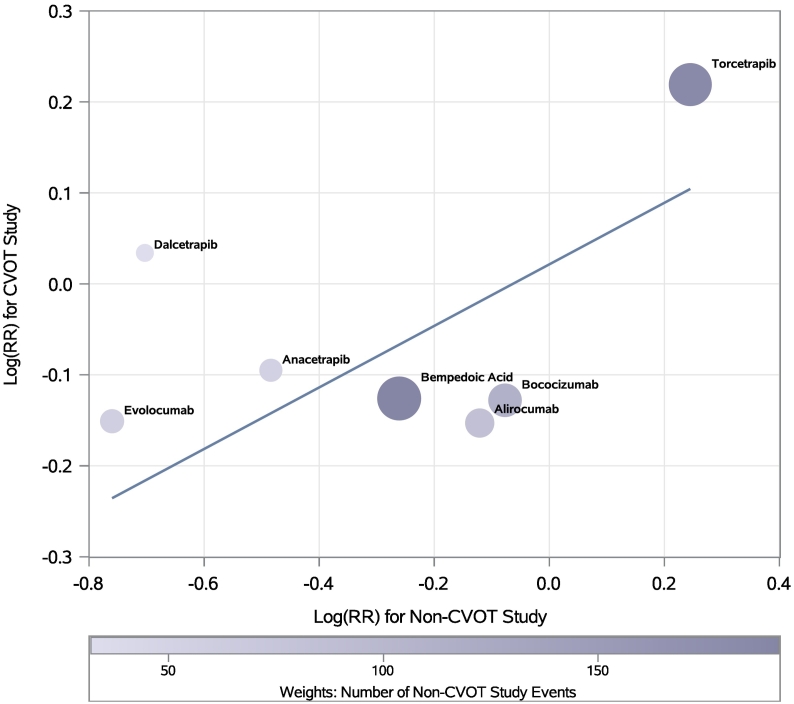
Weighted Linear Regression Between the log(*RR*)s from non-CVOTs and log(*HR*)s from CVOTs. HR- hazard ratio; RR- relative risk; drug names shown are those corresponding to Table 2 drug program listing.

There was a moderate correlation between the risk reductions or increases from the non-CVOTs and CVOTs: *r* = 0.69, *p* = 0.0893. The weighted linear regression (Fig. 1) illustrates how the treatment effect in non-CVOTs was generally greater (i.e., more negative in a log scale) than the treatment effect in the corresponding CVOT. This is reflected by the estimated slope (95% CI) for the regression line of 0.35 (0.02, 0.68), indicating that the expected log treatment effect in the CVOT is approximately one-third of what was observed in the non-CVOT. The figure also illustrates how the one program with a discordant non-CVOT and CVOT result, dalcetrapib, had fewer non-CVOT MACE events than any of the drugs compared.

## 4 Discussion

To our knowledge, this is the first systematic review of concordance between non-CVOT lipid study results that collected MACE safety data and their definitive counterpart MACE CVOT efficacy results. The results indicate that these data are predictive of the observed outcome in the pivotal CVOT. This result was unexpected to us as the non-CVOT trials are shorter in duration, enrolling lower risk subjects, with low power, and with a variable lag time to potential benefit for lipid-lowering drugs [10].

The weighted correlation and regression analyses showed that there is a moderate relationship between the non-CVOT and CVOT treatment effects. Importantly, the regression analysis suggested that the magnitude of the treatment effect in the CVOT was not necessarily well predicted from that observed in the non-CVOTs. This may be due to a lack of precision in the estimated treatment effects in non-CVOTs due to a limited number of MACE. In this study, the lack of concordance in the magnitude of the treatment effects were primarily due to dalcetrapib, anacetrapib, and evolocumab, which had treatment effects on a log scale that were several times larger in the non-CVOTs than in the corresponding CVOTs. However, these three programs also had the smallest numbers of non-CVOT MACE, which suggests lack of precision in the estimated treatment effects as a possible explanation for this discordance.

Many factors are clearly important in determining the progression of a drug to late-stage development and outcome of a lipid program CVOT, including, among others, the drug's mechanism of action (MOA), ability to reduce LDL-C/Apolipoprotein B, and prior proof of clinical benefit to reduce CV risk. In this regard, all three MOAs investigated here are capable, but do not always decrease LDL-C by upregulation of LDL receptors. This may explain the observed high rate of success in showing CVOT benefit [11]. The exception to this is dalcetrapib, which had accrued very few non-CVOT MACE events, showed no LDL-C lowering, and was stopped based on futility after a median of 31 months of follow-up. [12]

While there is always an urgency to complete drug development and approval as rapidly as possible, the results suggest the non-CVOT MACE data are informative and should be considered by sponsors, regulators, and other key stakeholders before launching into large, expensive and long-duration trials that may produce no benefit or even harm to participants. However, this is often not the case as non-CVOT MACE data typically become available only during CVOT execution, or not gathered at all, relying on the CVOT data alone when the decision to proceed is made.

If these findings are replicated, the value of this analysis lies in its ability to predict CVOT outcomes for cholesterol lowering drugs in development that are currently in progress and have or have not reported at least some phase 2/3 non-CVOT data while executing or planning to execute a CVOT study (see Table 3).

**Table 3:** Ongoing phase 3 programs with non-CVOT data and/or ongoing CVOT trials.

Drug	Phase 2/3 Non-CVOT Data	Ongoing CVOT Trial(s) [source CT.gov extracted 9-Jul-2025]	Estimated Primary Completion
**PCSK9**			
Inclisiran	3655 pts. (1827 pbo) treated for >18 mos, significantly reduced composite MACE [OR (95% CI): 0.74 (0.58–0.94)]. [45]	• ORION-4/ NCT03705234	7/2026
		• VICTORION-2P/ NCT05030428	10/2027
		• VICTORION-1 PREVENT/ NCT05739383	4/2029
lerodalcibep	• 2318 pts. (771 pbo) treated for 24 weeks MACE OR = 0.42 (0.19–0.94) • 1841 pts. (612 pbo) treated for 52 weeks MACE OR = 0.53 (0.30–0.92) [46]	Non listed as ongoing	n/a
Enlicitide; MK-0616	Phase 2b dose-ranging: 381 pts. (76 pbo) treated for 8 weeks +8 week follow-up. MACE safety reported for heterozygous FH study [12]. 1 CVD death in 202 MK-0616 pts., none in 101 pbo treated.	CORALreef Outcomes (TIMI 77) NCT06008756	11/2029
AZD-0780	Phase 2b dose-ranging: 428 pts. (87 pbo) treated for 12 weeks. No MACE safety reported [47].	AZURE-Outcomes NCT07000357	10/2029
**CETP Inhibitors**			
Obicetrapib	2882 (962 pbo) treated for 52 weeks, MACE OR = 0.77 (0.54, 1.11) [48]	PREVAIL NCT05202509	11/2026
**Lp(a) Inhibitors**			
Pelacarsen	Phase 2 dose-ranging: 286 pts. (47 pbo) for up to 1 year +16 weeks follow-up. No specific accounting of MACE safety [49]	Lp(a) HORIZON NCT04023552	2/2026
Olpasiran	Phase 2 dose ranging: 281 pts. (54 pbo) treated for 48 wk. + 24-week f/u. No specific accounting of MACE safety [50].	OCEAN(a) - Outcomes Trial NCT05581303	12/2026
lepodisiran	Phase 2 dose-ranging: 320 pts. (69 pbo) treated for 360 days. MACE-like SAE reports were 0 in pbo and 6 in active treatment (2 UA, 1 MI, 1 cardiac death, 1 cor. revasc) [51]	ACCLAIM-Lp(a) NCT06292013	3/2029
zerlasiran	Phase 2 dose-ranging: 178 pts. (47 pbo) treated for 48 wks + 12-week follow-up. MACE-like SAE reports were 0 in pbo and 3 MIs in active treatment [52]	Not yet initiated	
muvalapin	Phase 2 dose-ranging: 233 pts. (67 pbo) treated for 12 weeks. No specific accounting of MACE safety; 1 peripheral revascularization on pbo [53]	Not yet initiated	

CVOT = cardiovascular outcomes trial; cor. revasc = coronary revascularization; MACE = major adverse cardiovascular event; MI = myocardial infarction; pbo = placebo; pts. = patients; OR = odds ratio.; UA = unstable angina

As an illustration of the potential utility of the present analysis, first consider drugs with totally unproven clinical benefit, such as the Lp(a) programs that have a very strong biologic, experimental and epidemiologic basis for providing clinical benefit, but that has yet to be demonstrated. In this regard, Lp(a) drugs in late-stage development (pelacarsen, olpasiran, lepodisiran) have all initiated their CVOTs early in phase 3, likely due to the need to demonstrate clinical benefit in a CVOT prior to regulatory approval. However, the very limited non-CVOT data that has been published,does not show a clear trend for MACE imbalance in favor of the drug (see Table 3). We would recommend that, in these and similar instances, it is reasonable to consider alternatives to immediately initiating a traditional CVOT. For example, a CVOT can be delayed until such a time that approximately 100 patients have experienced a MACE in order to accrue sufficient non-CVOT data. This would not likely be a popular pathway, so alternatively, a phase 3 lipid program enhanced for high CV risk and MACE should be part of the lipid program design. Moreover, frequent monitoring of the CVOT by data monitoring committees (DMCs), including early and stringent futility analyses, could be performed. These DMC analyses and communication plans should be agreed to by sponsors and regulators. While usually for safety concerns, in some cases, a direct interface on evolving efficacy between a regulatory agency and a DMC may be appropriate.

In contrast to Lp(a) inhibition where no benefit has been previously demonstrated, CETP inhibitors have demonstrated some, albeit inconsistent, evidence of benefit in reducing CV events. Given this prior demonstration of benefit, it would seem appropriate for the non-CVOTs to progress while simultaneously initiating a CVOT. This is how the obicetrapib program was designed. Importantly, with the obicetrapib non-CVOTs now completed and the CVOT in progress, the sponsor is able to utilize blinded data to potentially modify the design and assumptions of the CVOT in a manner that preserves study integrity The non-CVOT data for obicetrapib showed a numeric imbalance in MACE (OR = 0.77) favoring the drug and suggesting a positive benefit for its PREVAIL CVOT, which was initiated early in phase 3 prior to the non-CVOT data being available. A positive outcome on PREVAIL would be a major success for the CETP inhibitor class that has seen three prior failures in CVOTs and one positive CVOT with anacetrapib and no regulatory approvals.

Finally, consider the case of a drug class that has consistently shown clinical benefit – the PCSK9 mAb inhibitors. In this case, we believe there is minimal value in non-CVOT data to predict CVOT outcome. In fact, the need for a CVOT in order to register PCSK9s for marketing approval can be argued. There are four PCSK9s currently in development; in three cases (inclisiran, lerodalcipeb, enlicitide) non-CVOT data have been partially reported, and in two (inclisiran, lerodalcipeb) showed a numeric imbalance in MACE in favor of the drug, suggesting a positive benefit would be seen in their CVOTs. Enlicitide recently reported a portion of its phase 3 lipid trial data. In the 52-week study of heterozygous FH, while no overall MACE data was published, 1 of 202 were reported to have died of ischemic stroke and cardiovascular death, while no deaths were reported in the 101 placebo-treated patients [13]. Inclisiran's CVOTs are anticipated to report out beginning in 2026 while lerodalcipeb's sponsor has not committed to a CVOT. AZD-0780's non-CVOT phase 2 MACE has not been reported but the sponsor has initiated its CVOT simultaneously with its phase 3 non-CVOT studies.

A potential limitation of using non-CVOTs to predict benefit in CVOT for therapeutics that reduce CV risk primarily through LDL-C lowering is the possibility of a “lag” in benefit, i.e., it may require 6–12 months of treatment before modification of risk attributable to LDL-C can be fully realized. This phenomenon has been observed in statin CVOTs [14], [15], [16], and more recently in PCSK9 inhibitor CVOTs [17]. This would suggest that any benefit of short-term treatment would not necessarily translate to a nominal reduction in risk in non-CVOTs with ≤1 year of follow-up. However, as previously discussed, the treatment effects in a subset of non-CVOTs were substantially greater than that observed in CVOTs. Importantly, the non-CVOTs with the greatest treatment effects also had the smallest numbers of events among the programs that were evaluated, ranging from 32 to 58. This raises the possibility that accruing more MACE could have yielded a more accurate estimate of efficacy, including whether there was evidence of a lag in benefit. This reinforces the recommendation of accruing approximately 100 MACE in non-CVOTs to facilitate a meaningful assessment of preliminary efficacy.

## 5 Conclusion

As clinical trial data for novel therapeutics are published, it is important to understand the implications of all data that are published, even that which is underpowered and not the primary objective of the trial. In conclusion, we observed in this retrospective study an unexpected predictability of non-CVOT data to be informative of a cholesterol lowering drug's CVOT outcome. Upcoming CVOTs over the next few years will determine if this analysis will be validated with a prospective ability to inform CVOT outcome.

## Prior presentations

None but submitted to National Lipid Association (non-oral) in June 2026, Chicago, IL, USA.

## CRediT authorship contribution statement

**Charles Shear:** Conceptualization, Data curation, Methodology, Validation, Writing – original draft, Writing – review & editing. **Michael H. Davidson:** Supervision, Writing – review & editing. **Marc Ditmarsch:** Conceptualization, Methodology, Writing – review & editing. **John J.P. Kastelein:** Conceptualization, Supervision, Writing – review & editing. **Michael Szarek:** Conceptualization, Formal analysis, Methodology, Writing – original draft, Writing – review & editing.

## Ethics approval

Ethical approval was not sought for the present study because all data used were previously published from deidentified studies that obtained ethics approval.

## Funding

Financial support for this work was provided in part by New Amsterdam Pharma. In sponsoring this work, no role was played in study design; in the collection, analysis and interpretation of data; in the writing of the report; and in the decision to submit the article for publication.

## Declaration of competing interest

**C Shear**: reports on previous employment with Merck, Pfizer Inc., CSL Ltd. and CIVI Biopharmaceuticals. He is currently a consultant for New Amsterdam Pharma Corp.

**M Davidson**: reports employment and shareholder at New Amsterdam Pharma.

**JPP Kastelein**: reports employment and shareholder at New Amsterdam Pharma.

**M Detmarsch**: reports employment and shareholder at New Amsterdam Pharma.

**M Szarek**: reports serving as a consultant or research support (or both) from Amarin, Lexicon, New Amsterdam, Novartis, Regeneron, Sanofi, Silence, and Tourmaline.

## Appendix A Supplementary data

Supplemental Table 1 CVOT and non-CVOT study details and use in analysis. Supplemental Table 1
