# Supplementary material for: Novel cholesterol lowering drugs: Can phase 2/3 clinical trial safety assessments predict cardiovascular event outcome trial efficacy?
Source: Am Heart J Plus. 2026 Jan 28;63:100728. doi: 10.1016/j.ahjo.2026.100728 (PMC12908039; doi:10.1016/j.ahjo.2026.100728)
Supplement: Supplemental Table 1 — CVOT and non-CVOT study details and use in analysis. [file mmc1.docx]

| **CETP Inhibitor: Evacetrapib** | | | | | | | | | | | | | | | | | |
| --- | --- | --- | --- | --- | --- | --- | --- | --- | --- | --- | --- | --- | --- | --- | --- | --- | --- |
| **CVOT** | **Lincoff et al 2017** | **6,038** | **6,054** | **2.3 yrs** |  | **x** | **x** | **x** | **X any** | **x** |  | **779** | **776** | **12.90** | **12.82** | **1.01** | **1.01 (0.91-1.11)** |
| non-CVOT | Nichols et al 2017 | 123 | 243 | 3 mo |  |  |  | x |  |  |  | 0 | 1 | 0.00 | 0.41 | not used |  |
| non-CVOT | Clin Trials.gov NCT02227784 | 247 | OLE | 12 mo |  |  | x |  |  |  | CAD | 2 | n/a | 0.81 | n/a | not used |  |
| non-CVOT | Teramoto et al 2018 | 53 | 96 | 3 mo |  |  |  |  |  |  | SAE* | 0 | 0 | 0.00 | 0.00 | not used |  |
| non-CVOT | Teramoto et al 2017 | 27 | 27 | 3 mo |  |  |  |  |  |  | SAE* | 0 | 0 | 0.00 | 0.00 | not used |  |
| **Non-CVOT Summary** | | **450** | **366** | **3-12 mo** |  |  |  |  |  |  |  | **2** | **1** | **0.44** | **0.27** | **Concordance not evaluated- see text** | |
| **CETP Inhibitor: Anacetrapib** | | | | | | | | | | | | | | | | | |
| **CVOT** | **HPS3/TIMI55–REVEAL Collab Grp 2017** | **15,225** | **15,224** | **4.1 yrs** | **x** |  | **x** |  | **X**  **any** |  |  | **1640** | **1803** | **10.77** | **11.84** | **0.91** | **0.91 (0.85-0.97)** |
| **CVOT** | [**HPS3/TIMI55–REVEAL Collab Grp 2021**](file:///C:\Users\ChuckShear\Desktop\Supplemental%20Table%201.xlsx#RANGE!_edn2) |  |  | **6.4 yrs** | **x** |  | **x** |  | **X**  **any** |  |  | **2146** | **2419** | **14.10** | **15.89** | **0.89** | **0.88(0.83-0.93)** |
| non-CVOT | [Kastelein et al 2015](file:///C:\Users\ChuckShear\Desktop\Supplemental%20Table%201.xlsx#RANGE!_edn3) | 808 | 804 | 76 wks |  | x | x | x |  | x |  | 16 | 21 | 1.98 | 2.61 | 0.76 |  |
| non-CVOT | [Ballantyne et al., 2017a](file:///C:\Users\ChuckShear\Desktop\Supplemental%20Table%201.xlsx#RANGE!_edn4) | 290 | 292 | 24 wks |  | x | x | x |  | x |  | 0 | 7 | 0.00 | 2.40 | 0.00 |  |
| non-CVOT | [Ballantyne et al., 2017b](file:///C:\\Users\\ChuckShear\\Desktop\\Supplemental%20Table%201.xlsx#RANGE!_edn5) | 305 | 154 | 24 wks |  | x | x | x |  | x |  | 2 | 2 | 0.66 | 1.30 | 0.50 |  |
| non-CVOT | [Teramoto et al., 2017b](file:///C:\\Users\\ChuckShear\\Desktop\\Supplemental%20Table%201.xlsx#RANGE!#REF!) | 204 | 103 | 24 wks |  | x | x | x |  | x |  | 1 | 0 | 0.49 | 0.00 | ND |  |
| non-CVOT | [Kastelein et al 2015](file:///C:\Users\ChuckShear\Desktop\Supplemental%20Table%201.xlsx#RANGE!#REF!) | 203 | 102 | 52 wks |  | x | x | x |  | x |  | 4 | 0 | 1.97 | 0.00 | ND |  |
| **Non-CVOT Summary** | | **1,810** | **1,455** | **3-18 mos** |  |  |  |  |  |  |  | **23** | **30** | **1.27** | **2.06** | **0.62** | **Concordant** |

| **CETP Inhibitor: Dalcetrapib** | | | | | | | | | | | | | | | | | | | |
| --- | --- | --- | --- | --- | --- | --- | --- | --- | --- | --- | --- | --- | --- | --- | --- | --- | --- | --- | --- |
| **CVOT** | [**Schwartz et al 2012**](file:///C:\Users\ChuckShear\Desktop\Supplemental%20Table%201.xlsx#RANGE!_edn1) | **7,938** | **7,933** | **2.6 yrs** | **x** |  | **x** | **X**  **isch** | |  | **x** | | **c.a.** | **656** | **633** | **8.26** | **7.98** | **1.04** | **1.04 (0.93-1.16)** |
| non-CVOT | [Luscher et al 2012](file:///C:\Users\ChuckShear\Desktop\Supplemental%20Table%201.xlsx#RANGE!_edn2) | 236 | 236 | 36 wks | x |  | x | X  isch | | X  any | x | | c.a. | 11 | 12 | 4.66 | 5.08 | 0.92 |  |
| non-CVOT | [Fayad et al 2011](file:///C:\Users\ChuckShear\Desktop\Supplemental%20Table%201.xlsx#RANGE!_edn3) | 63 | 65 | 24 mo | x |  | x | x isch | | X  any | x | | c.a. | 2 | 7 | 3.17 | 10.77 | 0.29 |  |
| non-CVOT | [Stein et al. 2009](file:///C:\Users\ChuckShear\Desktop\Supplemental%20Table%201.xlsx#RANGE!_edn4) | 218 | 74 | 12 wks | not pre-defined; one MI reported | | | | | | | | | 1 | 0 | 0.46 | 0.00 | not used |  |
| **Non CVOT Summary** | | **517** | **375** | **3-24 mos** |  |  |  |  | |  |  | |  | **13** | **19** | **2.51** | **5.07** | **0.50** | **Not concordant** |
| **CETP Inhibitor: Torcetrapib + atorvastatin** | | | | | | | | | | | | | | | | | | | |
| **CVOT** | [**Barter et al 2007**](file:///C:\Users\ChuckShear\Desktop\Supplemental%20Table%201.xlsx#RANGE!_edn1) | **7,533** | **7,534** | **1.5 yrs** | **x** |  | **x** | **x** | |  | **x** | |  | **464** | **373** | **6.16** | **4.95** | **1.24** | **1.25 (1.09-1.44)** |
| non-CVOT | [Nissen et al 2007](file:///C:\Users\ChuckShear\Desktop\Supplemental%20Table%201.xlsx#RANGE!_edn2) | 591 | 597 | 24 mos | x |  | x | x | |  | x | |  | 62 | 57 | 10.49 | 9.55 | 1.10 |  |
| non-CVOT | [Kastelein et al 2007](file:///C:\Users\ChuckShear\Desktop\Supplemental%20Table%201.xlsx#RANGE!_edn3) | 450 | 454 | 24 mos | SAEs of vascular origin | | | | | | | | | 24 | 11 | 5.33 | 2.42 | 2.20 |  |
| non-CVOT | [Bots et al 2007](file:///C:\Users\ChuckShear\Desktop\Supplemental%20Table%201.xlsx#RANGE!_edn4) | 377 | 375 | 22 mos | SAEs of vascular origin | | | | | | | | | 17 | 13 | 4.51 | 3.47 | 1.30 |  |
| **Non-CVOT Summary** | | **1,418** | **1,426** | **22-24 mos** |  |  |  |  | |  |  | |  | **103** | **81** | **7.26** | **5.68** | **1.28** | **Concordant** |
| **Bempedoic Acid** | | | | | | | | | | | | | | | | | | | |
| **CVOT** | [**Nissen et al 2023**](file:///C:\Users\ChuckShear\Desktop\Supplemental%20Table%201.xlsx#RANGE!_edn1) | **6,992** | **6,978** | **3.4 yrs** |  | **x** | **x** | **x** | **X**  **any** | | |  |  | **819** | **927** | **11.71** | **13.28** | **0.88** | **0.87 (0.79-0.96)** |
| non-CVOT high risk pool | FDA, 2019 NDA211616 | 2,009 | 999 | **51.8 wks** |  | x | x | x | x | | | x |  | 117 | 75 | 5.82 | 7.51 | 0.78 |  |
| non-CVOT low risk pool | FDA, 2019 NDA211616 | 415 | 198 |  | data not reported | | | | | | | | | | | | | |  |
| **Non-CVOT Summary** | | **2,424** | **1,197** | **12 mos** |  |  |  |  |  | | |  |  | 117 | 75 | 4.83 | 6.27 | **0.77** | **Concordant** |

| **PCSK9 Inhibitor: Evolocumab** | | | | | | | | | | | | | | | | | |
| --- | --- | --- | --- | --- | --- | --- | --- | --- | --- | --- | --- | --- | --- | --- | --- | --- | --- |
| **CVOT** | **Sabatine et al, 2017** | **13,784** | **13,780** | **2.2 yrs** |  | **x** | **x** | **x** | **X**  **any** | **x** |  | **1344** | **1563** | **9.75** | **11.34** | **0.86** | **0.85 (0.79-0.92)** |
| non-CVOT | [FDA, 2015. BLA Integrated Parent Studies, 2015](file:///C:\Users\ChuckShear\Desktop\Supplemental%20Table%201.xlsx#RANGE!_edn1) | 3,946 | 2,080 | 2.8 mos |  | x | x | x |  |  | TIA + HF | 25 | 9 | 0.63 | 0.43 | 1.46 | data not used duration too short |
| non-CVOT | [Sabatine et al 2015](file:///C:\Users\ChuckShear\Desktop\Supplemental%20Table%201.xlsx#RANGE!_edn2) | 1,314 | 655 | 12  mos |  | x | x | x |  |  | TIA + HF | 22 | 19 | 1.67 | 2.90 | 0.58 | Data -not used- as FDA pool used (next row) |
| non-CVOT | [FDA, 2015. BLA 125522 OSLER-1 and OSLER-2](file:///C:\Users\ChuckShear\Desktop\Supplemental%20Table%201.xlsx#RANGE!_edn3) | 2,976 | 1,489 | 11.1 mos | x |  | x | x  any | X  any | X | TIA+ HF | 28 | 30 | 0.94 | 2.01 | 0.47 |  |
| **NON-CVOT Summary** |  | **2,976** | **1,489** | **11.1 mos** |  |  |  |  |  |  |  | 28 | 30 | 0.94 | 2.01 | 0.47 | **Concordant** |
| **PCSK9 Inhibitor: Alirocumab** | | | | | | | | | | | | | | | | | |
| **CVOT** | **Schwartz et al 2018** | **9,462** | **9,462** | **2.8 yrs** | **x** |  | **x** | **x isch** |  | **x** |  | **903** | **1052** | 9.54 | 11.12 | **0.86** | **0.85(0.78-0.93)** |
| non-CVOT | [Jones et al 2016. 14 Ph 3 Trials- placebo controlled pool](file:///C:\Users\ChuckShear\Desktop\Supplemental%20Table%201.xlsx#RANGE!_edn1) | 2,318 | 1,174 | 1.2 yrs  (4029 pt-yrs/ 3340 pts.) |  |  |  |  |  |  |  | data not used as FDA pool available | | | | | |
| non-CVOT | [Jones et al 2016. 14 Ph 3 Trials- ezetimibe controlled pool](file:///C:\Users\ChuckShear\Desktop\Supplemental%20Table%201.xlsx#RANGE!_edn2) | 864 | 618 |  |  |  |  |  |  |  |  | data not used as FDA pool available | | | | | |
| non-CVOT | [FDA 2015. BLA 125559 global pool, medical review](file:///C:\Users\ChuckShear\Desktop\Supplemental%20Table%201.xlsx#RANGE!_edn3) | 3,182 | 1,792 | 27 weeks (eze pool) / 65 wks placebo pools | x |  | x | x isch- fatal and NF |  | x |  | 52 | 33 | 1.63 | 1.84 | 0.89 | 0.81 (0.52-1.25) |
| **Non-CVOT Summary** |  | **3,182** | **1,792** | **6-15.1 mos** |  |  |  |  |  |  |  | **52** | **33** | **1.63** | **1.84** | **0.89** | **Concordant** |

| **PCSK9 Inhibitor: Bococizumab** | | | | | | | | | | | | | | | | | |
| --- | --- | --- | --- | --- | --- | --- | --- | --- | --- | --- | --- | --- | --- | --- | --- | --- | --- |
| **CVOT SPIRE-1 and SPIRE-2** | **Ridker et al 2017a** | **13,720** | **13,718** | **7 mos (SPIRE-1) and 12 mos (SPIRE-2)** |  | **x** | **x** | **x** | **x** |  |  | **352** | **397** | **3.16** | **3.59** | **0.88** | 0.88 (0.76-1.02) rates are per 100 patient years |
| non-CVOT | Rider et al. 2017b | 2,377 | 2,058 | 12 to 52 weeks |  | x | x | x | x |  |  | 57 | 55 | 2.5 | 2.7 | 0.96 | rates are per 100 patient years |
| **non-CVOT Summary** |  | **2,377** | **2,058** | **12 to 52 weeks** |  | **x** | **x** | **x** | **x** |  |  | **57** | **55** | **2.5** | **2.7** | **0.96** | **Concordant** |

MACE= major adverse cardiovascular event; CHD= coronary heart disease; CVD= cardiovascular disease; NF-MI= non-fatal myocardial infarction; Cor Revasc= coronary revasc; Hosp UA/ACS= hospitalization for unstable angina or acute coronary syndrome; CVOT= cardiovascular outcomes trial; CAD= coronary artery disease; c.a.= cardiac arrest; ND= not defined; TIA= transient Ischemic attack; HF = hospitalization for heart failure

* SAE= serious adverse events; as there were none reported, no MACE is assumed
